# Seroprevalence and Associated Risk Factors for *Toxoplasma gondii* Infection in Healthy Blood Donors: A Cross-Sectional Study in Sonora, Mexico

**DOI:** 10.1155/2016/9597276

**Published:** 2016-06-30

**Authors:** Cosme Alvarado-Esquivel, Antonio Rascón-Careaga, Jesús Hernández-Tinoco, María Alba Guadalupe Corella-Madueño, Luis Francisco Sánchez-Anguiano, María Lourdes Aldana-Madrid, Edgar Velasquez-Vega, Trinidad Quizán-Plata, José Luis Navarro-Henze, Joel Alberto Badell-Luzardo, José María Gastélum-Cano, Oliver Liesenfeld

**Affiliations:** ^1^Biomedical Research Laboratory, Faculty of Medicine and Nutrition, Juárez University of Durango State, Avenida Universidad S/N, 34000 Durango, DGO, Mexico; ^2^Department of Chemical and Biological Sciences, University of Sonora, 83000 Hermosillo, SON, Mexico; ^3^Institute for Scientific Research “Dr. Roberto Rivera-Damm”, Juárez University of Durango State, Avenida Universidad S/N, 34000 Durango, DGO, Mexico; ^4^Department of Research and Postgraduate in Food, University of Sonora, 83000 Hermosillo, SON, Mexico; ^5^State Center for Blood Transfusion, Secretary of Health, 83000 Hermosillo, SON, Mexico; ^6^Mexican Institute of Social Insurance (IMSS), 83190 Hermosillo, SON, Mexico; ^7^Secretaría de Salud, 83270 Hermosillo, SON, Mexico; ^8^Institute for Microbiology and Hygiene, Charité Medical School, Campus Benjamin Franklin, Hindenburgdamm 27, 12203 Berlin, Germany

## Abstract

*Toxoplasma gondii* (*T*.* gondii*) can be transmitted by blood transfusion. We determined the prevalence of* T*.* gondii* infection in healthy blood donors in Hermosillo city, Mexico, and the association of infection with* T*.* gondii* with the sociodemographic, clinical, and behavioral characteristics of blood donors. Four hundred and eight blood donors who attended two public blood banks in Hermosillo city were examined for anti-*T*.* gondii* IgG and IgM antibodies by using enzyme-linked immunoassays. Of the 408 blood donors (mean age 31.77 ± 9.52; range 18–60 years old) studied, 55 (13.5%) were positive for anti-*T*.* gondii* IgG antibodies, and 12 (21.8%) of them were also positive for anti-*T*.* gondii* IgM antibodies. Multivariate analysis showed that seropositivity to* T*.* gondii* was associated with age (OR = 1.74; 95% CI: 1.03–2.94; *P* = 0.03) and tobacco use (OR = 2.09; 95% CI: 1.02–4.29; *P* = 0.04). Seropositivity to* T*.* gondii* was correlated with the number of pregnancies, deliveries, and cesarean sections. The seroprevalence of* T*.* gondii* infection in blood donors in Sonora is the highest reported in blood donors in northern Mexico so far. This is the first report of an association of* T*.* gondii* exposure and tobacco use. Further research to confirm this association is needed.

## 1. Introduction

The protozoan parasite* Toxoplasma gondii* (*T*.* gondii*) causes infections in humans and animals all around the world [1, 2]. Infection with* T*.* gondii* is often acquired by ingesting food or water contaminated with oocyst shed by cats or by eating raw or undercooked meat containing tissue cysts [2–4]. However,* T*.* gondii* infection can also be acquired by blood transfusion [5] and organ transplantation [6]. Dissemination of* T*.* gondii* occurs via blood flow to a large variety of body organs [7–9]. Primary infection during pregnancy may lead to congenital disease [2, 10]. Reactivation of* T*.* gondii* infection in immunocompromised patients may cause a life-threatening disease of the central nervous system [2, 11]. Signs and symptoms of toxoplasmosis usually include retinochoroiditis, enlargement of cervical lymph nodes, or encephalitis [1, 2]. In addition, infection with* T*.* gondii* has been linked to schizophrenia and other mental disorders [12, 13].

Very little is known about the seroepidemiology of* T*.* gondii* infection in blood donors in Mexico. We previously reported a 7.4% seroprevalence of* T*.* gondii* infection in blood donors in Durango city, Mexico [14], whereas two other studies of blood donors in central and southern Mexico reported seroprevalences of 29% [15] and 69% [16], respectively. There is no information about the magnitude of infection with* T*.* gondii* in blood donors in Hermosillo city, in the northwestern Mexican state of Sonora. Therefore, we sought to determine the seroprevalence of* T*.* gondii* infection in blood donors in Hermosillo city, Mexico, and the seroprevalence association with the sociodemographic, clinical, and behavioral characteristics of blood donors.

## 2. Materials and Methods

### 2.1. Study Design and Study Population

Through a cross-sectional study, we examined 408 blood donors who attended two blood banks (Mexican Institute of Social Insurance and State Center for Blood Transfusion of the Secretary of Health) in Hermosillo city, Mexico. Blood donors were enrolled consecutively. Blood sampling of blood donors was performed from November to December 2015. Inclusion criteria for enrollment of participants were (1) blood donors attending any of the participating blood banks; (2) those aged 18 years and older; and (3) those who voluntarily accepted to participate in the study. Of the 408 blood donors studied, 308 were enrolled in the blood bank of the Mexican Institute of Social Insurance and 100 in the State Center for Blood Transfusion.

### 2.2. Detection of Anti-*T*.* gondii* IgG and IgM Antibodies

Sera of blood donors were obtained by centrifugation of fresh whole blood and stored at −20°C until analyzed. Sera were analyzed for anti-*T*.* gondii* IgG antibodies by a commercially available enzyme immunoassay “*Toxoplasma* IgG” kit (Diagnostic Automation Inc., Woodland Hills, CA, USA). This test has a cut-off of 8 IU/mL of anti-*T*.* gondii* IgG antibodies for seropositivity. In addition, sera positive for anti-*T*.* gondii* IgG antibodies were further analyzed for anti-*T*.* gondii* IgM antibodies by a commercially available enzyme immunoassay “*Toxoplasma* IgM” kit (Diagnostic Automation Inc.). All tests were performed according to manufacturer's instructions, and positive and negative controls were included in each assay.

### 2.3. Sociodemographic, Clinical, and Behavioral Data

Through a face-to-face interview using a standardized questionnaire, we obtained the sociodemographic, clinical, and behavioral data of blood donors. Sociodemographic data included age, gender, birth place, residence, educational level, occupation, and socioeconomic status. Clinical data included health status, presence or history of lymphadenopathy, frequent abdominal pain or headache, dizziness, impairment of memory, reflexes, hearing, or vision, and history of surgery, blood transfusion, or organ transplantation. In women, obstetric history was obtained. Behavioral data included contact with animals, cleaning cat excrements, traveling, soil contact, type of flooring at home, frequency of eating away from home (in restaurants and fast food outlets), eating raw or undercooked meat, washing hands before eating, history of sexual promiscuity, and tobacco, alcohol, or drug use. In addition, behavioral items included frequency of meat consumption, type of meat consumed (pork, lamb, beef, goat, boar, chicken, turkey, rabbit, deer, squirrel, horse, fish, and others), consumption of dried or cured meat (chorizo, ham, sausages, or salami), beef intestine or stomach, animal brains, unwashed raw vegetables or fruits, and unpasteurized milk or untreated water.

### 2.4. Statistical Analysis

Data was analyzed with the aid of the software Epi-Info version 7 and SPSS version 15 (SPSS Inc., Chicago, IL, USA). For calculation of the sample size we used a reference seroprevalence of 7.4% [14] as expected frequency of the factor under study, 15,000 as the population size from which the sample was selected, confidence limits of 2.6%, and a confidence level of 95%. The result of the calculation was 380 subjects. To avoid bias in the statistical analysis, blood donors with missing data were excluded from the study. For comparison of the frequencies among groups we used Pearson's chi square test and the Fisher exact test (when cells values were small). The association between the characteristics of blood donors and* T*.* gondii* seropositivity was assessed by bivariate and multivariate analyses. Only variables with a *P* value < 0.05 obtained in the bivariate analysis were included in the multivariate analysis. Odds ratio (OR) and 95% confidence interval (CI) were calculated by multivariate analysis using the Enter method. Statistical significance was set at a *P* value < 0.05.

### 2.5. Ethical Aspects

This study was approved by the Institutional Ethics Committee of the University of Sonora, Mexico. All participants were informed about the purpose and procedures of the survey, and a written informed consent was obtained from all of them.

## 3. Results

Of the 408 blood donors (mean age 31.77 ± 9.52; range 18–60 years old) 55 (13.5%) were positive for anti-*T*.* gondii* IgG antibodies. Of these 55 seropositive donors, 32 (58.2%) had IgG levels higher than 150 IU/mL, 3 (5.4%) between 100 and 150 IU/mL, and 20 (36.4%) between 12 to 97 IU/mL. Seroprevalence of anti-*T*.* gondii* IgG antibodies in blood donors from the Mexican Institute of Social Insurance (43/308: 14.0%) was similar (*P* = 0.61) to the one (12/100: 12.0%) in the State Center for Blood Transfusion. Of the 55 IgG seropositive blood donors, 12 (21.8%) were also positive for anti-*T*.* gondii* IgM antibodies.

Concerning the correlation of* T*.* gondii* IgG seropositivity with the sociodemographic characteristics of blood donors, bivariate analysis showed a number of characteristics with a *P* value < 0.05 including age, residence, educational level, and occupation. Other sociodemographic characteristics of blood donors, that is, gender, birth place, and socioeconomic status, showed *P* values > 0.05 by bivariate analysis. A selection of sociodemographic characteristics and their correlation with* T*.* gondii* seropositivity is shown in Table 1.

Of the clinical characteristics of blood donors, seropositivity to* T*.* gondii* did not correlate (*P* > 0.05) with health status, presence or history of lymphadenopathy, frequent abdominal pain or headache, dizziness, impairment of memory, reflexes, hearing, or vision, and history of surgery, blood transfusion, or organ transplantation. In women, seropositivity to* T*.* gondii* correlated with number of pregnancies, deliveries, and cesarean sections (*P* < 0.05).  Table 2 shows the correlation of* T*.* gondii* seropositivity and the obstetric data of the female blood donors studied.

With respect to the correlation of seropositivity to* T*.* gondii* and behavioral characteristics of blood donors, a number of characteristics showed *P* values < 0.05 by bivariate analysis including cleaning cat excrement, tobacco consumption, and type of flooring at home. Other behavioral characteristics, that is, contact with animals, traveling, soil contact, frequency of eating away from home, eating raw or undercooked meat, washing hands before eating, history of sexual promiscuity, alcohol consumption, and drug use, showed *P* values > 0.05 by bivariate analysis.  Table 3 shows a selection of behavioral characteristics of blood donors and their correlation with* T*.* gondii* seropositivity. Further analysis of sociodemographic and behavioral characteristics of blood donors by multivariate analysis showed that seropositivity to* T*.* gondii* was associated with age (OR = 1.74; 95% CI: 1.03–2.94; *P* = 0.03) and tobacco use (OR = 2.09; 95% CI: 1.02–4.29; *P* = 0.04) (Table 4).

The correlation of IgM seropositivity with the blood bank and the sociodemographic, clinical, and behavioral characteristics of the 55 blood donors with IgG seropositivity to* T*.* gondii* was also analyzed. The variables blood bank, age, no cats in the neighborhood, and turkey meat consumption showed association with IgM seropositivity by bivariate analysis (Table 5). Other variables examined in this survey did not show a correlation with IgM seropositivity (*P* > 0.05).

## 4. Discussion

The seroepidemiology of infection with* T*.* gondii* in blood donors in Mexico has been scantly studied. The present study aimed to know the seroprevalence and associated risk factors of infection with* T*.* gondii* in a sample of blood donors who attended two blood banks in the northwestern Mexican city of Hermosillo. We found a 13.5% seroprevalence of* T*.* gondii* infection in the blood donors studied. This seroprevalence is higher than the 7.4% seroprevalence of* T*.* gondii* infection reported in blood donors in the northern Mexican city of Durango [14]. In contrast, the seroprevalence found in blood donors of Hermosillo city is lower than the 29% seroprevalence reported in blood donors in Jalisco state [15] and the 69% seroprevalence reported in a control group of blood donors in Yucatán state [16] in central and south Mexico, respectively. The seropositivity rate for* T*.* gondii* in blood donors in Hermosillo is lower than seroprevalences reported in blood donors in other countries in South America where seroprevalences between 20 and 79% have been reported [17, 18]. Seroprevalences reported in blood donors in other countries were 40% in Saudi Arabia [19], 59.6% in Egypt [20], 20.3% in India [21], 42.9% in New Zealand [22], and 32.1% in the Czech Republic [23]. On the other hand, the seroprevalence found in blood donors in Hermosillo is comparable with seroprevalences of* T*.* gondii* infection reported in blood donors in Iran (19.3%) [24], Taiwan (9.3%) [25], Turkey (19.5%) [26], and Thailand (9.6%) [27] and even lower than that reported in blood donors in Shijiazhuang area, China (4.83%) [28]. Differences in seroprevalences of* T*.* gondii* infection in blood donors among countries might be due to differences in the behavioral characteristics of blood donors, that is, extent of* T*.* gondii* exposure, or differences in the environment. Hermosillo city has a hot desert climate and seroprevalence of* T*.* gondii* infection is usually lower in dry climates than in humid climates [1]. Seroprevalence in blood donors does not reflect the seroprevalence in general population because blood donors are usually healthy and have a limited range of age whereas healthy and ill individuals with a wide range of age can exist in the general population.

We searched for sociodemographic, clinical, and behavioral factors of blood donors associated with* T*.* gondii* seropositivity. Multivariate analysis showed that age was associated with* T*.* gondii* seroprevalence, with the highest rate of positivity in the age group of 31–50 years. This result is consistent with that in a study of blood donors in Durango, Mexico, where the highest seroprevalence of* T*.* gondii* infection was found in the 45–60-year-old group [14]. Similarly, in a study of blood donors in Thailand, researchers found the highest rate of* T*.* gondii* seropositivity in the age groups of 20–30 and 31–40 years old [27]. Intriguingly, multivariate analysis also showed that* T*.* gondii* seropositivity was associated with tobacco use in blood donors in Hermosillo. To the best of our knowledge, this is the first report of an association of tobacco use and seropositivity to* T*.* gondii*. It is not clear why tobacco users had a higher seroprevalence of* T*.* gondii* infection than blood donors without tobacco use. It is possible that tobacco users might acquire* T*.* gondii* infection by carrying the parasite from the hands to the mouth while smoking. In principle, washing hands before smoking is not commonly done; therefore, handling the cigarette and touching the lips with unwashed hands while smoking might contribute to acquiring* T*.* gondii* infection. Sharing the cigarette among people might potentially increase this risk of infection since cigarette is touched with more hands of more people. The hand-cigarette-mouth route has been linked to* Salmonella* infection [29]. Further research to determine the role of smoking in* T*.* gondii* transmission is needed. On the other hand, infection with* T*.* gondii* may lead to changes in behavior [30, 31]. It is unknown whether infection with* T*.* gondii* may lead to a desire to smoke. Of note, both infection with* T*.* gondii* and smoking are linked to dopamine release. Experimental infections with* T*.* gondii* in mice resulted in an increase of dopamine release in brain [32]. Similarly, nicotine elicits dopamine release in the ventral striatum that is a substance underlying tobacco smoking behaviors and dependence [33]. It is also unclear whether behavioral changes induced by nicotine may favor an increase in the exposure to* T*.* gondii*. Tobacco smoking has been associated to a number of health outcomes including infections, that is, hepatitis C virus [34], postoperative infections after dental implants [35], or spine surgeries [36]. However, the mechanisms involved in the association between infection and tobacco smoking are poorly understood [37].

Seropositivity to* T*.* gondii* was correlated with the number of pregnancies, deliveries, and cesarean sections. This correlation might be related to an increasing seroprevalence of* T*.* gondii* infection with age. The older the women, the higher the likelihood to accumulate obstetric events. In addition, it is also possible that a history of blood transfusion, a known risk factor for* T*.* gondii* infection [5], could have contributed to infection in some women with obstetric events.

Concerning the seropositivity to anti-*T*.* gondii* IgM antibody in anti-*T*.* gondii* IgG seropositive blood donors, bivariate analysis showed a higher IgM seropositivity rate in blood donors attended in the State Center for Blood Transfusion than in those attended in the Mexican Institute of Social Insurance. It is possible that differences in the characteristics of blood donors among blood banks might explain the difference in the seroprevalences. IgM seropositivity was also associated with age and turkey meat consumption. Turkeys can be infected by* T*.* gondii*. In a study in Egypt, a 29.4% seroprevalence of* T*.* gondii* infection in turkeys was found [38]. In addition, in two previous studies in Mexico,* T*.* gondii* seropositivity was associated with consumption of turkey meat in pregnant women [39] and in rural general population [40]. In the present study, IgM seropositivity was higher in subject without cats in the neighborhood than those with cats in the neighborhood. These results can be interpreted as a higher risk of* T*.* gondii* infection by eating tissue cysts of turkey meat than by ingestion of oocysts shed by cats in the blood donors studied. On the other hand, the presence of IgM positive blood donors could suggest the existence of more recent infections. However, IgM positivity should be interpreted with care because* T*.* gondii* commercial IgM diagnostic test kits can yield a number of false positive results [41].

DNA of* T*.* gondii* has been found in 1.9% of IgM seropositive blood donors [24]; therefore, the use of blood from* T*.* gondii* IgM positive blood donors might represent a risk for* T*.* gondii* infection.

The present study has some limitations. The sample size of one blood bank was small. The small number of IgM seropositive cases did not allow us to perform further multivariate analysis with this infection marker. In addition, we were unable to perform molecular detection of* T*.* gondii* in samples of blood donors. Results from DNA detection may further help to determine the risk for* T*.* gondii* infection by blood transfusion. Further research with a large sample size of blood donors and with the use of molecular methods for detection of* T*.* gondii* should be conducted.

## 5. Conclusions

The seroprevalence of* T*.* gondii* infection in blood donors in Hermosillo city in the northwestern Mexican state of Sonora is the highest reported in blood donors in northern Mexico so far. Seropositivity to* T*.* gondii* was linked to obstetric events and age. This is the first report of an association of* T*.* gondii* exposure and tobacco use. Further research to confirm this association is needed.

## Figures and Tables

**Table 1:** Sociodemographic characteristics of blood donors and prevalence of *T. gondii* infection.

Characteristic	Number	Prevalence of *T. gondii* infection		*P* value
		Number	%	
Age groups (years)				
30 or less	206	15	7.3	0.001
31–50	186	38	20.4	
>50	16	2	12.5	

Gender				
Male	334	47	14.1	0.45
Female	74	8	10.8	

Birth place				
Sonora state	366	49	13.4	0.59
Other Mexican states	39	5	12.8	
Abroad	3	1	33.3	

Residence area				
Urban	373	45	12.1	0.008
Suburban	27	9	33.3	
Rural	8	1	12.5	

Educational level				
No education	10	4	40.0	0.004
1 to 6 years	48	6	12.5	
7–12 years	216	36	16.7	
>12 years	134	9	6.7	

Occupation				
Laborer^a^	328	51	15.5	0.01
Nonlaborer^b^	80	4	5.0	

Socioeconomic level				
Low	195	32	16.4	0.24
Medium	212	23	10.8	
High	1	0	0.0	

^a^Laborer: construction, professional, business, agriculture, factory worker, and others.

^b^Nonlaborer: housewife, student, or no occupation.

**Table 2:** Bivariate analysis of obstetric data and infection with *T. gondii* in female blood donors.

Characteristic	Subjects tested	Prevalence of *T. gondii *infection		*P* value
	Number	Number	%	
Pregnancies				
None	21	1	4.8	0.01
One	6	0	0	
Two	17	1	5.9	
Three	13	1	7.7	
Four	7	2	28.6	
Five	1	1	100	

Deliveries				
None	36	4	11.1	0.03
One	8	1	12.5	
Two	13	0	0	
Three	6	0	0	
Four	1	0	0	
Five	1	1	100	

Cesarean sections				
None	43	2	4.7	0.01
One	10	0	0	
Two	4	1	25	
Three	8	3	37.5	

Stillbirths				
None	64	6	9.4	1.00
One	1	0	0	

Miscarriages				
None	59	6	10.2	1.00
One	6	0	0	

**Table 3:** Bivariate analysis of selected putative risk factors for infection with *T. gondii *in blood donors.

Characteristic	Subjects tested	Prevalence of* T. gondii* infection		*P* value
	Number	Number	%	
Cats in the neighborhood				
Yes	265	39	14.7	0.31
No	143	16	11.2	

Cleaning cat excrement				
Yes	42	10	23.8	0.03
No	366	45	12.3	

Pork meat consumption				
Yes	269	39	14.5	0.40
No	139	16	11.5	

Sheep meat consumption				
Yes	24	1	4.2	0.22
No	384	54	14.1	

Pigeon meat consumption				
Yes	2	1	50.0	0.25
No	406	54	13.3	

Duck meat consumption				
Yes	2	1	50.0	0.25
No	406	54	13.3	

Quail meat consumption				
Yes	4	1	25.0	0.44
No	404	54	13.4	

Venison consumption				
Yes	40	3	7.5	0.24
No	368	52	14.1	

Iguana meat consumption				
Yes	2	1	50.0	0.25
No	406	54	13.3	

Fish consumption				
Yes	244	39	16.0	0.07
No	164	16	9.8	

Alcohol consumption				
Yes	171	23	13.5	0.98
No	237	32	13.5	

Tobacco consumption				
Yes	61	14	23.0	0.01
No	347	41	11.8	

Drug use				
Yes	45	10	22.2	0.06
No	363	45	12.4	

Soil contact				
Yes	61	11	18.0	0.25
No	347	44	12.7	

Washing hands before eating				
Yes	390	50	12.8	0.08
No	18	5	27.8	

Floor at home				
Ceramic or wood	204	26	12.7	0.03
Concrete	203	28	13.8	
Soil	1	1	100.0	

**Table 4:** Multivariate analysis of selected characteristics of blood donors and their association with *T. gondii* infection.

Characteristic	Odds ratio	95% confidence interval	*P* value
Age	1.74	1.03–2.94	0.03
Suburban residence	1.56	0.78–3.12	0.20
Education	0.76	0.48–1.19	0.23
Laborer	0.38	0.13–1.15	0.08
Cleaning cat excrement	0.47	0.20–1.09	0.08
Flooring at home	0.83	0.43–1.58	0.57
Tobacco use	2.09	1.02–4.29	0.04

**Table 5:** Correlation of seroprevalence of anti-*T. gondii* IgM antibodies with a selection of characteristics of 55 IgG seropositive blood donors.

Characteristic	Number	Prevalence of anti-*T. gondii* IgM antibodies		*P* value
		Number	%	
Blood bank				
Mexican Institute of Social Insurance	43	6	14.0	0.01
State Center for Blood Transfusion	12	6	50.0	

Age groups (years)				
30 or less	15	7	46.7	0.01
31–50	38	4	89.5	
>50	2	1	50.0	

Cats in the neighborhood				
Yes	39	4	10.3	0.003
No	16	8	50.0	

Turkey meat consumption				
Yes	9	5	55.6	0.01
No	46	7	15.2	

## References

[1] Dubey J. P. (2010). *Toxoplasmosis of Animals and Humans*.

[2] Montoya J. G., Liesenfeld O. (2004). Toxoplasmosis. *The Lancet*.

[3] Torrey E. F., Yolken R. H. (2013). *Toxoplasma* oocysts as a public health problem. *Trends in Parasitology*.

[4] Jones J. L., Dubey J. P. (2012). Foodborne toxoplasmosis. *Clinical Infectious Diseases*.

[5] Figueroa Damián R. (1998). Risk of transmission of infectious diseases by transfusion. *Ginecología y obstetricia de México*.

[6] Barsoum R. S. (2006). Parasitic infections in transplant recipients. *Nature Clinical Practice Nephrology*.

[7] Unno A., Suzuki K., Xuan X., Nishikawa Y., Kitoh K., Takashima Y. (2008). Dissemination of extracellular and intracellular *Toxoplasma gondii* tachyzoites in the blood flow. *Parasitology International*.

[8] Harker K. S., Ueno N., Lodoen M. B. (2015). *Toxoplasma gondii* dissemination: a parasite's journey through the infected host. *Parasite Immunology*.

[9] Ueno N., Lodoen M. B. (2015). From the blood to the brain: avenues of eukaryotic pathogen dissemination to the central nervous system. *Current Opinion in Microbiology*.

[10] Oz H. S. (2014). Maternal and congenital toxoplasmosis, currently available and novel therapies in horizon. *Frontiers in Microbiology*.

[11] Machala L., Kodym P., Malý M., Geleneky M., Beran O., Jilich D. (2015). Toxoplasmosis in immunocompromised patients. *Epidemiologie, Mikrobiologie, Imunologie*.

[12] Sutterland A. L., Fond G., Kuin A. (2015). Beyond the association. *Toxoplasma gondii* in schizophrenia, bipolar disorder, and addiction: systematic review and meta-analysis. *Acta Psychiatrica Scandinavica*.

[13] Alvarado-Esquivel C., Urbina-Álvarez J. D., Estrada-Martínez S. (2011). *Toxoplasma gondii* infection and schizophrenia: a case control study in a low *Toxoplasma* seroprevalence Mexican population. *Parasitology International*.

[14] Alvarado-Esquivel C., Mercado-Suarez M. F., Rodríguez-Briones A. (2007). Seroepidemiology of infection with *Toxoplasma gondii* in healthy blood donors of Durango, Mexico. *BMC Infectious Diseases*.

[15] Galván Ramirez M. D. L. L., Covarrubias X., Rodríguez R., Troyo R., Alfaro N., Correa D. (2005). *Toxoplasma gondii* antibodies in Mexican blood donors. *Transfusion*.

[16] Góngora-Biachi R. A., González-Martínez P., Castro-Sansores C. (1998). Antibodies against *Toxoplasma gondii* in patients with HIV in Yucatán. *Revista de Investigacion Clinica*.

[17] Coêlho R. A. L., Kobayashi M., Carvalho L. B. (2003). Prevalence of IgG antibodies specific to *Toxoplasma gondii* among blood donors in Recife, Northeast Brazil. *Revista do Instituto de Medicina Tropical de Sao Paulo*.

[18] Zamorano C. G., Contreras M. C., Villalobos S., Sandoval L., Salinas P. (1999). Seroepidemiological survey of human toxoplasmosis in Osorno, Region X, Chile, 1998. *Boletín chileno de parasitología*.

[19] Makki S. M., Abdel-Tawab A. H. (2010). Anti-*Toxoplasma gondii* antibodies among volunteer blood donors in eastern Saudi Arabia. *Journal of the Egyptian Society of Parasitology*.

[20] Elsheikha H. M., Azab M. S., Abousamra N. K., Rahbar M. H., Elghannam D. M., Raafat D. (2009). Seroprevalence of and risk factors for *Toxoplasma gondii* antibodies among asymptomatic blood donors in Egypt. *Parasitology Research*.

[21] Sundar P., Mahadevan A., Jayshree R. S., Subbakrishna D. K., Shankar S. K. (2007). *Toxoplasma* seroprevalence in healthy voluntary blood donors from urban Karnataka. *Indian Journal of Medical Research*.

[22] Zarkovic A., MacMurray C., Deva N., Ghosh S., Whitley D., Guest S. (2007). Seropositivity rates for *Bartonella henselae*, *Toxocara canis* and *Toxoplasma gondii* in New Zealand blood donors. *Clinical & Experimental Ophthalmology*.

[23] Svobodová V., Literák I. (1998). Prevalence of IgM and IgG antibodies to *Toxoplasma gondii* in blood donors in the Czech Republic. *European Journal of Epidemiology*.

[24] Sarkari B., Shafiei R., Zare M., Sohrabpour S., Kasraian L. (2014). Seroprevalence and molecular diagnosis of *Toxoplasma gondii* infection among blood donors in southern Iran. *Journal of Infection in Developing Countries*.

[25] Chiang T.-Y., Hsieh H.-H., Kuo M.-C. (2012). Seroepidemiology of *Toxoplasma gondii* Infection among Healthy Blood Donors in Taiwan. *PLoS ONE*.

[26] Yazar S., Eser B., Yay M. (2006). Prevalence of anti-*Toxoplasma gondii* antibodies in Turkish blood donors. *Ethiopian Medical Journal*.

[27] Pinlaor S., Ieamviteevanich K., Pinlaor P., Maleewong W., Pipitgool V. (2000). Seroprevalence of specific total immunoglobulin (Ig), IgG and IgM antibodies to *Toxoplasma gondii* in blood donors from Loei Province, Northeast Thailand. *Southeast Asian Journal of Tropical Medicine and Public Health*.

[28] Yang S.-J., Song R.-H. (2012). Seroprevalence of *Toxoplasma gondii* antibodies in healthy voluntary blood donors from Shijiazhuang area. *Chinese Journal of Schistosomiasis Control*.

[29] El-Tras W. F., Tayel A. A., Samir A. (2010). Potential zoonotic pathways of Salmonella enteritidis in laying farms. *Vector Borne and Zoonotic Disease*.

[30] Kamerkar S., Davis P. H. (2012). *Toxoplasma* on the brain: understanding host-pathogen interactions in chronic CNS infection. *Journal of Parasitology Research*.

[31] McConkey G. A., Martin H. L., Bristow G. C., Webster J. P. (2013). *Toxoplasma gondii* infection and behaviour—location, location, location?. *Journal of Experimental Biology*.

[32] Gatkowska J., Wieczorek M., Dziadek B., Dzitko K., Dlugonska H. (2013). Sex-dependent neurotransmitter level changes in brains of *Toxoplasma gondii* infected mice. *Experimental Parasitology*.

[33] Cosgrove K. P., Esterlis I., Sandiego C., Petrulli R., Morris E. D. (2015). Imaging tobacco smoking with pet and spect. *Current Topics in Behavioral Neurosciences*.

[34] Waziry R., Jawad M., Ballout R. A., Al Akel M., Akl E. A. (2016). The effects of waterpipe tobacco smoking on health outcomes: an updated systematic review and meta-analysis. *International Journal of Epidemiology*.

[35] Keenan J. R., Veitz-Keenan A. (2016). The impact of smoking on failure rates, postoperative infection and marginal bone loss of dental implants. *Evidence-Based Dentistry*.

[36] Saeedinia S., Nouri M., Azarhomayoun A. (2015). The incidence and risk factors for surgical site infection after clean spinal operations: a prospective cohort study and review of the literature. *Surgical Neurology International*.

[37] Calvo M., Laguno M., Martínez M., Martínez E. (2015). Effects of tobacco smoking on HIV-infected individuals. *AIDS reviews*.

[38] Harfoush M., Tahoon A. E.-N. (2010). Seroprevalence of *Toxoplasma gondii* antibodies in domestic ducks, free-range chickens, turkeys and rabbits in Kafr El-Sheikh Governorate Egypt. *Journal of the Egyptian Society of Parasitology*.

[39] Alvarado-Esquivel C., Sifuentes-Álvarez A., Narro-Duarte S. G. (2006). Seroepidemiology of *Toxoplasma gondii* infection in pregnant women in a public hospital in northern Mexico. *BMC Infectious Diseases*.

[40] Alvarado-Esquivel C., Cruz-Magallanes H. M., Esquivel-Cruz R. (2008). Seroepidemiology of *Toxoplasma gondii* infection in human adults from three rural communities in Durango state, Mexico. *Journal of Parasitology*.

[41] Dhakal R., Gajurel K., Pomares C., Talucod J., Press C. J., Montoya J. G. (2015). Significance of a positive *Toxoplasma* immunoglobulin M test result in the United States. *Journal of Clinical Microbiology*.

